# Socio-demographic factors, informal payments and satisfaction with childbirth in the Hungarian context

**DOI:** 10.1186/s12884-025-07521-3

**Published:** 2025-04-08

**Authors:** Orsolya Udvari, Ivett Szalma

**Affiliations:** 1 Corvinus University of Budapest, Budapest, Hungary; 2 Hungarian Demographic Research Institute, Budapest, Hungary; 3 HUN-REN Centre for Social Sciences, Budapest, Hungary

**Keywords:** Childbirth satisfaction, Socio-demographic factors, Informal payments, Obstetric care, Healthcare inequalities

## Abstract

**Objective:**

This study aims to investigate the impact of socio-demographic factors on childbirth satisfaction in Hungary, with a particular focus on the role of informal payments. While previous research has extensively examined maternal satisfaction, the specific influence of socio-demographic characteristics on childbirth experiences remains insufficiently explored, particularly in Hungary, where informal payments and a dual healthcare system coexist. By addressing this gap, the study seeks to provide a deeper understanding of the factors that shape birth satisfaction.

**Methods:**

This study examines satisfaction with childbirth using a representative sample of the Hungarian adult population surveyed between February and April 2024. Descriptive statistics (chi-square test) and factor analysis were applied to understand perceptions of quality obstetric care, including responses from childless women and men on general attitudes towards obstetric services (*N* = 1360). Logistic regression examined socio-demographic predictors of satisfaction with obstetric care among mothers and fathers who evaluated their partners’ experiences (*N* = 772).

**Results:**

Findings underscore the significance of financial factors in childbirth care. Individuals with lower education levels often consider informal payments and private doctors essential for quality care (χ² = 18.0, *p* < 0.05). Factor analysis revealed two key dimensions: financial and competency. Emphasis on financial aspects correlated with dissatisfaction (Financial components, Factor 1: OR = 0.74, 95%, *p* < 0.05) while prioritizing competency was linked to higher satisfaction (Competence, Factor 2: OR = 1.54, 95%, *p* < 0.01). The percentage of women dissatisfied with their childbirth experience was 13.8%, compared to 6.1% of men.

**Conclusion:**

Efforts to reduce reliance on informal payments and enhance equitable access to high-quality obstetric care are critical for improving childbirth satisfaction in Hungary.

**Supplementary Information:**

The online version contains supplementary material available at 10.1186/s12884-025-07521-3.

## Introduction

Childbirth experience and satisfaction have been shown to have short- and long-term effects on the health and well-being of mothers and their families. This is why organizations such as the WHO emphasize the importance of positive childbirth experiences [1]. Research suggests that positive childbirth experiences can promote feelings of empowerment, achievement, and increased self-esteem following childbirth. Conversely, negative or traumatic childbirth experiences have been associated with adverse outcomes, including postpartum mental health disorders such as postpartum depression, anxiety, and post-traumatic stress disorder (PTSD) [2–4]. Such experiences may also impact maternal-infant bonding, breastfeeding, and decisions about future pregnancies [5, 6].

The multidimensional nature of childbirth satisfaction has been widely researched, with findings suggesting it is influenced by various factors, including demographic characteristics, birth preparation, perceived control, pain management, delivery mode, and support from healthcare professionals and family members [7–11]. Medical interventions, such as emergency procedures (e.g. high-risk pregnancies) and pain relief, play a critical role in shaping childbirth experiences [6, 9, 12–14].

Additionally, socio-demographic factors, including socioeconomic status, ethnicity, and education are crucial determinants of maternal outcomes. Women from lower socioeconomic backgrounds and vulnerable groups, such as ethnic minorities and teenage mothers, face higher risks of negative maternal and perinatal outcomes, including postpartum depression, preterm birth, and neonatal mortality [15–18]. The relationship between educational attainment and childbirth satisfaction remains inconclusive in the literature. While some studies suggest that women with higher educational backgrounds report greater satisfaction with their overall experience [19–21], others argue that highly educated women have higher expectations that are often unmet, leading to lower satisfaction levels compared to women with lower levels of education [22, 23].

Support (cognitive and emotional) provided by a companion chosen by the woman has been showed to have a positive effect on satisfaction [10]. Fathers’ presence during labor has also been shown to enhance maternal satisfaction, primarily through emotional support and mediation [24, 25]. While fathers often report positive experiences, their involvement is sometimes limited by inadequate information or preparation, leading to feelings of uncertainty [26]. Fathers who were present at birth generally reported a positive experience, although they often felt uncertain about the situation [27, 28]. However, the role of fathers in influencing overall childbirth satisfaction remains understudied. In Hungary, where the healthcare system and social inequalities may uniquely shape maternal experiences, there is limited research on the influence of socio-demographic factors and paternal involvement on childbirth satisfaction.

This study aims to address this knowledge gap by examining general attitudes toward the factors considered essential for obtaining quality obstetric care in Hungary. The study addresses the following questions: What factors affect the Hungarian population’s perception of quality obstetric care? How do socio-demographic factors influence satisfaction with childbirth in Hungary? What roles do informal payments play in childbirth satisfaction?

## Dual healthcare system and the role of informal payments

Hungary’s healthcare system combines state-funded public services and a growing private sector, resulting in a dual practice system that significantly impacts patient satisfaction. Public healthcare is free for individuals insured through social security contributions, which are typically linked to employment. However, the private healthcare system often provides higher quality and faster services, particularly in obstetric care [29]. This “hybridization” of healthcare has exacerbated social inequalities, as access to better care often depends on financial resources.

A further country-specific and regional (Central and Eastern Europe) factor contributing to healthcare inequalities was the practice of informal payments (IPs), also known as gratitude payments or gratitude money, which originated in the early 1950s. This “specific manifestation of the informal economy found in the healthcare sector” served as a means for patients to pay to healthcare professionals for (or hope to secure) better care and treatment within the public healthcare system [30]. In the Hungarian obstetric care IPs allowed women to have and hire a “chosen/private obstetrician” who would provide care during both prenatal visits and obstetric care during labour, in exchange for these payments. In addition, IPs have been used indirect mechanisms to keep the income of health care professionals consistently low [31].

The practice of IPs exists in several parts of the world, such as India [32]. However, in the Central and Eastern European region, IPs have distinct socio-historical characteristics. Comparative studies covering Bulgaria, Hungary, and Ukraine [33, 34], as well as Romania, Bulgaria, and Moldova [35], have revealed similar mechanisms across countries, with some specific differences. The proportion of healthcare expenditures attributed to IPs varies significantly across the region, accounting for approximately 1.5–4.6% of total health spending in Hungary, 0.3–0.5% in Poland, and around 2% in Bulgaria [34]. Cross-country comparisons indicate that public perceptions of IPs vary significantly: in some countries—especially in Poland but also in Bulgaria—attitudes toward informal payments are less favorable than in others, such as Hungary and Ukraine [33].

In 2021, the Hungarian government implemented reforms aimed at eliminating IPs and addressing healthcare inequalities. Informal payments were criminalized, and healthcare workers’ employment conditions were revised to improve transparency and income parity [31, 36]. Similar reform attempts have been made in the region to eradicate corruption in public institutions and increase transparency, including within the healthcare system. For instance, since 1999, Poland’s healthcare system has shifted toward providing egalitarian access while also implementing market-type mechanisms [37]. One of the main aims of these early reforms was to eliminate IPs. Other studies have shown that private sector payments have replaced IPs in order to receive more attentive care [38]. Another example: in March 2018, Romania implemented a significant one-time wage raise for public healthcare professionals, which affected the prevalence of informal payments [39]. Some of these reforms were not directly aimed at eliminating informal payments but were part of broader anti-corruption policy reform trends [40]. The Hungarian reforms also included measures to restrict the movement between private and public healthcare systems, meaning that if a patient chooses private care for a medical issue, they must continue to receive care privately for that issue. However, to benefit from public antenatal care, individuals are required to have valid healthcare insurance, while delivery (an acute situation) is supported by the National Health Insurance Fund even without valid health insurance. Despite these efforts, the 2022 Eurobarometer survey revealed that many Hungarians still consider it acceptable to offer gifts or favors to access public services, indicating lingering cultural acceptance of informal practices [41].

IPs are part of out-of-pocket payments, direct payments “for healthcare goods and services from the household primary income or savings, where the payment is made by the user at the time of the purchase of goods or the use of the services” [42]. This category also covers spending on pharmaceuticals which means that separating the two dimensions is challenging. Measuring and obtaining data on informal payment expenses remains inconclusive because of its sensitive nature, as people might not report it because of trust and protection issues, similar to corruption [43, 44].

## Methods

### Study sample and design

The survey was conducted between February and April 2024 on a sample of 1,506 individuals, we employed a cross-sectional study design. The research was funded by the MTA TK Lendület Research Group on Reproductive Decisions. The sample was nationally representative by age, gender, and type of settlement. The representativeness of the sample was ensured through post-stratification weighting using the 2022 Census data.

Data collection was carried out by Panelstory. Participants were recruited through a hybrid method: 66.9% (*N* = 1,008) completed an online questionnaire, while 33.1% (*N* = 498) participated through in-person interviews, ensuring that individuals without internet access were included. The research focused on reproductive decisions, covering topics such as abortion and its consequences, contraception usage, fertility treatments, and knowledge about adoption. The questionnaire was supplemented with a section focusing on childbirth experiences.

### Outcome variable

The questionnaire included seven items that examined who might receive better services. These were the following: those in a better financial situation, those with adequate knowledge about hospital procedures, those who communicate effectively with hospital staff, those who give IPs to hospital staff, those whose relatives are present during childbirth, those who belong to an ethnic or national minority, and those who have a privately hired obstetrician.

The respondents could answer these items on a four-point scale, where 1 meant “not worse,” 2 meant “no, the same,” 3 meant “yes, better,” and 4 meant “yes, much better.” We asked everyone these questions, including those who have not had children, as they may still have perceptions or knowledge about obstetric care.

Satisfaction with childbirth was measured with the following item: “How satisfied were you with the obstetric care during the birth of your (youngest) child?” Four response options were provided: very satisfied, rather satisfied, rather dissatisfied, and completely dissatisfied. We deliberately phrased the question in a gender-neutral manner to also capture men’s satisfaction with the childbirth experience.

### Independent variables

We included the following independent variables in the analysis: the respondent’s gender (male, female), educational attainment (low, medium, high), financial status (struggles with income, does not struggle with income), the father’s presence (present or not in the delivery room during the childbirth), and the year of childbirth (before 1990, between 1990 and 1999, between 2000 and 2009, between 2010 and 2019, or after 2020). The variable respondent’s age was excluded from the analysis due to its high correlation with the year of birth of the youngest child.The description of these variables is presented in Table 1.

**Table 1:** Description of variables included in the analysis

*Variables*	*Values*	*n*	*Percentage*
Satisfaction	Satisfied	642	89.2
	Not satisfied	78	10.8
Gender	Male	275	38.0
	Female	449	62.0
Education	Low	236	32.6
	Middle	328	45.4
	Higher	159	22.0
Financial status	Struggles	172	23.8
	Does not struggle	550	76.2
Presence of father in the delivery room	Was not present	512	70.7
	Was present	212	29.3
Year of giving birth	After 2020	77	10.6
	2010–2019	187	25.8
	2000–2009	107	14.8
	1990–1999	129	17.8
	Before 1990	224	30.9

### Data analysis

**Fig. 1:**
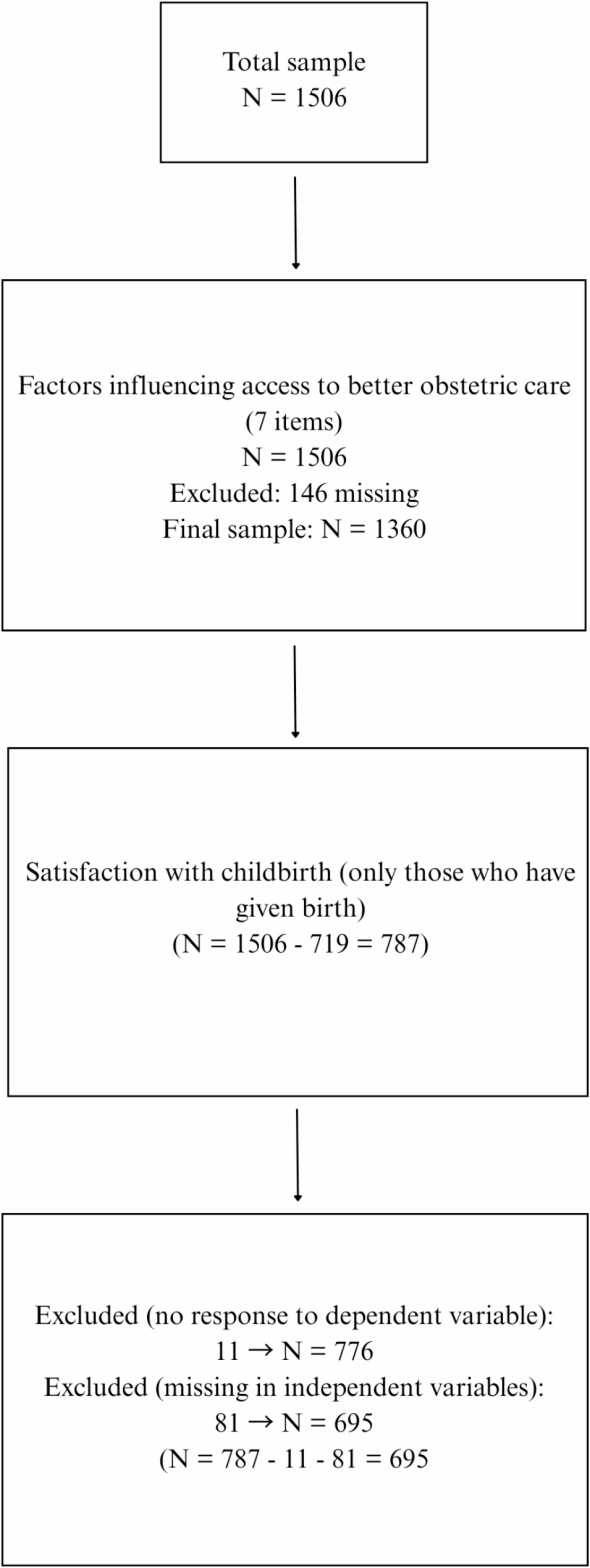
Study population: a study flowchart with number of included/excluded cases

We conducted statistical analyses using Stata v18. Figure 1 shows the sample size for the analyses performed. Categorical variables were coded, described by proportions, and comparisons were made using the chi-square test. Factor analysis was employed to construct an index reflecting respondents’ perceptions of the factors influencing the quality of obstetric care. Sampling adequacy was assessed using the Kaiser-Meyer-Olkin (KMO) measure, which yielded a value of 0.869, indicating adequate sampling for factor analysis. Bartlett’s test of sphericity was also conducted to examine the presence of significant correlations among the variables. A significant Bartlett’s test result (*p* < 0.05) indicated the presence of correlations among variables, justifying the use of factor analysis. Following factor analysis, we employed logistic regression to examine associations with birth satisfaction. Although ordinal logistic regression could have been applied to the four-level variable, the highly skewed distribution of the dependent variable—where over 88% of respondents selecting one of the two most positive categories—suggested that an ordinal logistic regression would likely encounter estimation issues due to sparse data in the lower categories. To ensure the stability and interpretability of the model, we opted for a binary outcome. Odds ratios and 95% confidence intervals were calculated for each variable. A p-value of < 0.05 was considered statistically significant.

## Results

### Sample characteristics

A total of 1,506 individuals participated in the survey, and the sample was representative of the Hungarian population with 42.3% men and 57.6% women. Consequently, women were slightly over-represented, which may have been influenced by the research topic. The average age of participants was 47.3 years. In terms of educational attainment, 27.2% had primary or vocational education, 47.8% had secondary education, and 25% had a university degree.

Out of the sample of 1,506, 787 individuals had given birth, which means 52% of the sample. However, out of the 787 individuals, 92 did not answer one of the questions we examined, so they were excluded from the sample. Thus, the sample distribution resulted in 61.5% women and 38.5% men. Given that the childlessness rate is higher among men than among women in Hungary [45]; and women are more likely to respond to questions related to childbirth, they are over-represented in our subsample. The mean ages of male and female participants were 53.8 and 52.9 years, respectively.

As for satisfaction with obstetric care, 1 in 10 respondents reported dissatisfaction. Table 2 shows the distribution of responses according to the gender of the respondents. Satisfaction with childbirth was measured with the following item: “How satisfied were you with the obstetric care during the birth of your (youngest) child?”

**Table 2:** Satisfaction with obstetric care by gender (%)

	Completely dissatisfied	Rather not satisfied	Rather satisfied	Very satisfied
**Women**	3.2	10.6	39.9	46.4
**Men**	0	6.1	46.1	47.8

The gender distribution indicates that women report significantly higher dissatisfaction than men: nearly 1 in 7 women (13.8%) reported dissatisfaction, while among men, this rate was approximately half as much (6.1%). The sample includes 32.6% with low education, 45.4% with secondary education, and 22% with a degree. Financially, 76.2% manage comfortably, while 23.8% face difficulties. Less than 30% of fathers were present at childbirth, though this has increased over time, rising from 3.1% before 1990 to 54.6% after 2020.

Given that the study covers multiple birth cohorts, we adopt a longitudinal approach in assessing satisfaction with obstetric care.

### Factors influencing access to better obstetric care services

In the questionnaire, we examined which resources are considered necessary for better obstetric care across seven dimensions: financial means, knowledge, communication, informal payments, presence of relatives, ethnic background, and having a private (chosen) obstetrician. First, we analysed the full sample to assess perceptions of these factors and then explored whether significant differences emerged based on gender, educational level, and age group (See Table 3).

**Table 3:** Factors influencing the quality of obstetric care by gender, age, and educational level

Variables	Worse	Same	Somewhat better	Much better	Gender	Education	Age group
Financial	4.5	27.5	43.3	24.7	0.8	18.0**	25.0***
Knowledge	4.2	40.7	39.2	16.0	0.2	14.0*	17.7**
Communication	3.8	34.4	42.3	19.5	2.8	10.8	13.4*
Gratitude money	5.3	22.1	41.8	30.7	0.5	20.8**	20.4**
Relatives	6.1	39.0	37.4	17.5	1.4	12.2	21.1**
Ethnic background	22.6	47.7	19.8	10.0	5.3	28.3***	21.1**
Private obstetrician	5.4	17.1	38.1	39.3	3.3	21.3**	23.7***

Notes: Note: *p* < 0.05, *p* < 0.01, *p < 0.001: Pearson Chi-square (χ²) test was used to analyse the cross-tabulations, and the significance levels indicated were used to determine the statistical significance of the results

Nearly 40% of respondents believe that individuals with a private obstetrician receive significantly better care, while an additional 38% think they receive somewhat better care. The fewest respondents (10%) consider ethnic background as a factor influencing the quality of care. Following the provision of private obstetrician services, most respondents (72%) perceive IPs and gratitude money as a factor that leads to better care, followed by those who believe that individuals in better financial situations receive superior care (68%). Similarly, approximately 60% of the sample believes that adequate knowledge of the hospital system and effective communication can lead to somewhat or significantly better care. About 55% of the sample considers the presence of relatives as a factor that contributes to better care.

The last three columns display values from the Pearson Chi-square test, segmented by gender, educational level, and age group. Interestingly, there are no significant differences between genders regarding access to better services for any of the items. However, significant differences based on educational attainment are observed in almost all dimensions, except for the presence of relatives. Individuals with lower educational levels deemed every dimension (excluding the presence of relatives) to be significantly more important compared to those with higher educational levels. Regarding age groups, the 40–59 age group found every dimension to be significantly more important than the other two age groups (18–39 and 60+).

### Factor analysis

Based on these seven variables, we attempted to establish a factor structure. The items appeared to separate into two dimensions: financial factors and competency-related factors. However, we had to exclude the ethnicity variable from this structure, as it did not align with either factor. Each entry in Table 4 shows the relative effect of each factor on the potential for better obstetric care that respondents could receive; these values are referred to as factor loadings. Table 4 also indicates the communality of each variable, which refers to the proportion of variance in the dependent variable explained by the attitudinal statements.

**Table 4:** Factor loadings and communality values for attitudinal variables included in sorted, varimax rotated factor matrix, by variable (*N* = 1360)

Item	Financial components (Factor 1)	Competence (Factor 2)	Communality values
Who is in a better financial situation	0.65	n.a.	0.64
Who has adequate knowledge of hospital procedures	n.a.	0.78	0.72
Who communicates effectively with the institution’s staff	n.a.	0.75	0.69
Who gives gratuity money to the hospital staff	0.82	n.a.	0,78
Who has a relative present during childbirth	n.a.	0.63	0.61
Who has a private obstetrician	0.74	n.a.	0.66
Eigenvalue	2,54	1,01	n.a.
Variance explained	42,3%	16,8%	n.a.

Note: n.a.=not applicable

The first factor, which links access to better obstetric services with financial components, explains 88% of the variance in access to better obstetric care. The second factor, which strongly correlates with statements linking access to better care with competency-related items, explains about 8% of the total variance. This suggests that respondents prioritize financial factors over competencies when it comes to accessing better obstetric care in Hungary.

### Logistic regression

We examined satisfaction with obstetric care among parents, comparing the opinions of men and women. The estimated factor scores were incorporated into a logistic regression analysis along with selected socio-demographic factors, such as gender, education, subjective financial status, presence of fathers at the birth of the child, and year of delivery.

The logistic regression analysis (Table 5) revealed a significant gender effect: women reported lower satisfaction with obstetric care compared to men, with an Odds Ratio (OR) of 0.47 (95% CI: 0.267–0.840), indicating that women were less likely to be satisfied with care than men, a difference that was statistically significant (*p* < 0.01). Education also had a significant impact on satisfaction. Individuals with higher educational attainment were less satisfied compared to those with lower education (OR for higher education = 0.40, 95% CI: 0.190–0.830), which suggests that dissatisfaction is more prevalent among those with higher education, potentially reflecting differing expectations or experiences with obstetric care.

Those facing financial struggles reported significantly lower satisfaction with obstetric care (OR = 0.49, 95% CI: 0.276–0.862, *p* < 0.01). This indicates that financial difficulties are associated with significantly lower satisfaction levels with obstetric care.

**Table 5:** Logistic regression model of satisfaction levels with obstetric care, *N* = 695

Predictor	Odd ratios	95% CI
**Gender** (female)	0.47**	0.267–0.840
**Education** (ref. low)	1	
Middle	0.65	0.334–1.249
Higher	0.40**	0.190–0.830
**Financial status** (ref. make ends meet)	1	
Struggles financially	0.49**	0.276–0.862
**Father’s presence**	1.69	0.876–3.243
**Year of giving birth** (ref. before 1990)	1	
After 2020	0.22***	0.094–0.516
2010–2019	1.32	0.542–3.188
2000–2009	0.63	0.270–1.477
1990–1999	0.52	0.246–1.077
**Financial components (Factor 1)**	0.74*	0.550–0.990
**Competence (Factor 2)**	1.54**	1.084–2.195

Notes: *p < 0.05, **p < 0.01, ***p < 0.001, *N* = 687, Pseudo *R*^2^ = 0.099

The presence of fathers at childbirth, while associated with increased satisfaction (OR = 1.69, 95% CI: 0.876–3.243), was not statistically significant, likely due to the small sample size in this category. However, the trend suggests that father involvement may positively influence satisfaction.

Year of childbirth also significantly impacted satisfaction levels. Those who gave birth after 2020 reported notably lower satisfaction levels (OR = 0.22, 95% CI: 0.094–0.516, *p* < 0.001), indicating a marked decline in satisfaction compared to those who gave birth before 1990.

Two additional factors—financial components (Factor 1) and perceived competence (Factor 2)—were significant. Economic difficulties were associated with lower satisfaction (OR = 0.74, 95% CI: 0.550–0.990, *p* < 0.05), while those who rated competence of care higher were more satisfied (OR = 1.54, 95% CI: 1.084–2.195, *p* < 0.01).

## Discussion

Our research examined a relatively underexplored area, satisfaction with childbirth in Hungary. This is particularly significant in light of the 2021 legislative changes that abolished informal payments, along with the option to choose a private obstetrician. Nevertheless, our 2024 survey revealed that the Hungarian population still places significant importance on the financial dimension, especially the choice of private obstetricians and the phenomenon of informal payments. We did not find significant gender differences, but in terms of educational attainment, respondents with lower levels of education and those in the 40–59 age group tend to believe that access to better care is facilitated by informal payments and private obstetrician consultations. Individuals with lower educational levels also believe that those with an ethnic background receive better obstetric care. This is surprising, as previous research has highlighted those individuals with an ethnic background, particularly Roma, receive poorer and discriminatory care [17]. Our factor analysis revealed two dimensions based on the six variables: a financial dimension and a competency dimension.

When we examined a narrower sample– those who had childbirth experience, either directly or as fathers whose children were born– we found that 1 in 10 respondents expressed dissatisfaction with obstetric care, which is roughly consistent with the general 8–10% rate of negative childbirth feedback [6, 16, 46, 47]. However, if we consider that other studies have focused only on women, the dissatisfaction rate in our study is higher, with 1 in 7 women reporting dissatisfaction.

The results of the logistic regression analysis also highlight that women are more dissatisfied with childbirth than men. This is surprising, because some research shows that men had similar experiences as their partners [28]. Further research is needed to explore gender differences in satisfaction with obstetric care. Furthermore, the higher someone’s level of education, the greater the likelihood of dissatisfaction, and those who consider the financial components more important are also more dissatisfied. Previous research has highlighted the importance of educational attainment, with some studies reporting effects consistent with our findings [22, 23]. However, our results contradict those of Konieczka et al., who observed that female respondents with higher levels of education exhibited significantly higher childbirth satisfaction—particularly regarding the alignment of expectations with the birth plan [19]. This positive association between maternal education and childbirth satisfaction was further supported by research from Jafari et al. [20] and Dolatian et al. [21], which demonstrated that childbirth satisfaction increased with higher levels of mothers’ education. Thus, there are contradictory findings in the literature regarding the role of educational attainment.

In the Hungarian case, the lower level of satisfaction observed among highly educated women may be explained by the fact that they are generally more familiar with healthcare processes, more aware of their rights and options, and enter the healthcare system with specific expectations (e.g., after attending childbirth preparation classes). However, the Hungarian healthcare system may not be equipped to meet these expectations. In contrast, less informed or lower-educated patients tend to have less clearly defined expectations, making them less likely to experience disappointment and more inclined to perceive their experiences as normal.

Our examination of both recent and earlier childbirth experiences revealed that those who gave birth after 2020 were less satisfied with obstetric care compared to those who gave birth before 1990. It is noteworthy that those who gave birth after 2020 were the least satisfied. This may be due to the fact that Hungarian society was not prepared for the removal of informal payments. However, this effect could also stem from the COVID-19 pandemic and the state of emergency declared in the spring of 2020. Numerous studies have investigated the impact of the pandemic on childbirth satisfaction, with many findings indicating that COVID-19 increased dissatisfaction in most settings, primarily due to unpredictability and heightened stress [48–51]. This, in turn, had a significant negative impact on maternal well-being, notably contributing to a rise in postpartum depression. However, some research—such as studies conducted in Italy [52] and Russia [53]—has found no differences in overall satisfaction between pre-COVID-19 and during COVID-19 birthing cohorts.

Therefore, it is crucial to distinguish the extent to which the decline in satisfaction after 2020 can be attributed to the abolition IPs versus COVID-19 related restrictions. During the COVID-19 pandemic, Romania (22%) and Bulgaria (19%) recorded the highest incidence of bribery in the public health sector among all EU member countries [54]. In the Romanian context, Mosca et al. found that patient-reported informal payments decreased during the ‘low payment’ period (before the 2018 wage increase for healthcare workers) and further declined during the ‘high payment’ period (after the 2018 wage increase). Additionally, the share of respondents willing to report informal payments increased during the “high pay” period, suggesting a greater willingness to express dissatisfaction with health services and IPs, though this trend slowed during the first lockdown in 2020. Contrarily, some argue that the pandemic may have acted as a catalyst for IPs [54]. Thus, in the Hungarian context, the literature suggests—and further investigation is warranted—to assess the extent to which underlying dissatisfaction during COVID has been compounded by restrictions on informal payments, given that demand for such payments may have increased (for quicker services, for example), but legislative regulations prevented their emergence due to concerns over legal consequences. However, other studies have highlighted the role of marketization, that private sector payments have replaced IPs in order to receive more attentive care [38].

The state of emergency affected hospital visits as well, although the father could be present during childbirth even during the COVID-19. At the same time, a positive trend can be observed, as in the last one and a half decades, the presence of fathers at childbirth has increased: since 2010, more than half of fathers have been present at births. Although we did not find a statistically significant effect, this may be due to the limited sample size. Future studies with larger samples could provide more conclusive evidence on the impact of paternal presence on childbirth satisfaction.

### Strengths and limitations

One of the key strengths of this study is that it was conducted using a representative sample, which allows for generalizability of the findings to the broader population. Additionally, the longitudinal aspect of the study enabled us to analyse data over time, shedding light on the potential negative impacts of recent legislative changes on obstetric care. This approach provided valuable insights into how legislative shifts in the healthcare system may affect individuals’ satisfaction with childbirth care in Hungary.

A significant limitation of the study is the small sample size when focusing on individuals who gave birth in recent years. This limited sample size affects our ability to examine the influence of factors such as paternal presence on satisfaction with obstetric care with sufficient statistical power. Additionally, having data from both partners would allow for a more comprehensive analysis. Measuring satisfaction levels for both women and men regarding the same childbirth experience would provide a more nuanced understanding of the dynamics of obstetric care satisfaction. Moreover, we did not assess the effect of the mode of delivery, which previous research suggests might be an important factor in analysing birth experience [55–58].

## Conclusions

Our results suggest that public attitudes toward informal payments and private obstetricians remain unchanged, indicating that legislative reforms alone have not been sufficient. Awareness-raising campaigns are necessary to clarify that financial status should not influence obstetric care, especially since lower-educated individuals and those aged 40–59 perceive a strong link between financial means and care quality. Moreover, those who prioritize competency report higher satisfaction, highlighting the need for effective communication and professional knowledge in obstetric care. Notably, individuals who gave birth after the 2020 legislative change were significantly less satisfied with their care. Further research is needed to understand this trend, particularly given its overlap with the COVID-19 pandemic. Our findings emphasize persistent inequalities in access to obstetric care, underscoring the need for targeted awareness campaigns on the elimination of informal payments.

## Electronic supplementary material

Below is the link to the electronic supplementary material.

Supplementary Material 1

## Data Availability

The survey was specifically designed and developed by the authors for the purposes of this research. The English version of the questionnaire is provided as a supplementary file (Supplementary File 1). The datasets used and/or analyzed during the current study are available from the corresponding author upon reasonable request.
