# Supplementary material for: Socio-demographic factors, informal payments and satisfaction with childbirth in the Hungarian context
Source: BMC Pregnancy Childbirth. 2025 Apr 8;25:409. doi: 10.1186/s12884-025-07521-3 (PMC11980149; doi:10.1186/s12884-025-07521-3)
Supplement: Supplementary file 1 — Supplementary Material 1 [file 12884_2025_7521_MOESM1_ESM.docx]

# Scientific research on having children

Dear Respondent,

This questionnaire was commissioned by the “Momentum” Reproductive Sociology Research Group, funded by the Hungarian Academy of Sciences. The work of the research group can be followed at <https://reprosoc.tk.hu>.

The researchers aim to explore the opinions of Hungarians regarding childbearing, whether they are young or old, women or men. The research is led by Ivett Szalma, PhD, Research Professor at the HUN-REN Centre for Social Sciences. If you have any questions about the research, please contact [szalma.ivett@tk.hu](mailto:szalma.ivett@tk.hu).

The topics are wide-ranging, and we ask you to answer this questionnaire as honestly as possible and to the best of your knowledge. If you feel uncomfortable completing it, you may stop at any time.

Participation is voluntary, but successful responses will be rewarded with a 2000 HUF EMAG voucher, which will be automatically sent to your email address within a few hours after submitting the questionnaire. Your response is anonymous: your answers cannot be linked to you personally.

The length of the survey will vary depending on the individual, but it will take approximately 30 minutes to complete. Thank you for your time in completing the questionnaire.

Best regards,

The Panelstory Team

First, we ask you about your views on having children in general.

1. How much do you agree with the following statements?

# Having children is necessary for a woman to live a fulfilled life.

How much do you agree with this?

| 1 | 2 | 3 | 4 | 5 |
| --- | --- | --- | --- | --- |
| I don’t agree at all | I rather disagree | I agree and disagree | I rather agree | I totally agree |

- 1. **It** **is** **right** **for** **the** **husband** **to** **put** **work** **first** **and** **the** **wife** **to** **put** **home** **and** **children** **first,** **even** **if** **they** **both** **work.**

How much do you agree with this?

| 1 | 2 | 3 | 4 | 5 |
| --- | --- | --- | --- | --- |
| I don’t agree at all | I rather disagree | I agree and disagree | I rather agree | I totally agree |

# Having children is important because Hungary’s population is declining.

How much do you agree with this?

| 1 | 2 | 3 | 4 | 5 |
| --- | --- | --- | --- | --- |
| I don’t agree at all | I rather disagree | I agree and disagree | I rather agree | I totally agree |

# Climate change will mean that the standard of living of children born today will be much lower than ours.

How much do you agree with this?

| 1 | 2 | 3 | 4 | 5 |
| --- | --- | --- | --- | --- |
| I don’t agree at all | I rather disagree | I agree and disagree | I rather agree | I totally agree |

# A single mother can raise her child just as well as two parents together.

How much do you agree with this?

| 1 | 2 | 3 | 4 | 5 |
| --- | --- | --- | --- | --- |
| I don’t agree at all | I rather disagree | I agree and disagree | I rather agree | I totally agree |

# A single father can raise his child as well as two parents together.

How much do you agree with this?

| 1 | 2 | 3 | 4 | 5 |
| --- | --- | --- | --- | --- |
| I don’t agree at all | I rather disagree | I agree and disagree | I rather agree | I totally agree |

# Pets are considered as part of the family.

How much do you agree with this?

| 1 | 2 | 3 | 4 | 5 |
| --- | --- | --- | --- | --- |
| I don’t agree at all | I rather disagree | I agree and disagree | I rather agree | I totally agree |

- 1. **A** **pet** **can** **play** **as** **important** **a** **role** **in** **a** **person's** **life** **as** **a** **child.**

How much do you agree with this?

| 1 | 2 | 3 | 4 | 5 |
| --- | --- | --- | --- | --- |
| I don’t agree at all | I rather disagree | I agree and disagree | I rather agree | I totally agree |

- 1. **A** **couple** **of** **two** **women** **can** **raise** **a** **child** **just** **as** **well** **as** **a** **couple** **of** **a** **man** **and** **a** **woman.**

How much do you agree with this?

| 1 | 2 | 3 | 4 | 5 |
| --- | --- | --- | --- | --- |
| I don’t agree at all | I rather disagree | I agree and disagree | I rather agree | I totally agree |

- 1. **A** **couple** **of** **two** **men** **can** **raise** **a** **child** **just** **as** **well** **as** **a** **couple** **of** **a** **man** **and** **a** **woman.**

How much do you agree with this?

| 1 | 2 | 3 | 4 | 5 |
| --- | --- | --- | --- | --- |
| I don’t agree at all | I rather disagree | I agree and disagree | I rather agree | I totally agree |

# Having children is a duty to society.

How much do you agree with this?

| 1 | 2 | 3 | 4 | 5 |
| --- | --- | --- | --- | --- |
| I don’t agree at all | I rather disagree | I agree and disagree | I rather agree | I totally agree |

# How much would you say it would be good or bad for the Hungarian population to come to live here from other countries to offset the depopulation?

Indicate where you rank on the scale, where 0 means it would be very bad and 10 means it would be very good.

0 1 2 3 4 5 6 7 8 9 10

It would be very bad It would be very good

# In general, how much does the fact that people from other countries come to live here hurt or help Hungary's economy?

Indicate where your opinion ranks on the scale, where 0 means it does a lot of harm and 10 means it does a lot of good.

0 1 2 3 4 5 6 7 8 9 10

it’ very harmful it uses a lot

# Would you say that the cultural life of Hungary in general is harmed or enriched by people from other countries coming to live here??

Indicate where your opinion ranks on the scale, where 0 means very harmful and 10 means very enriching.

0 1 2 3 4 5 6 7 8 9 10

very harmful very enrich

# How important do you consider these to be for someone to be called Hungarian?

- 1. **Hungary** **should** **be** **his/her** **birthplace.**

How important is it for someone to be called Hungarian?

1 2 3 4

not at all important rather not important quite important very important

# He/she should have Hungarian ancestors or relatives.

How important is it for someone to be called Hungarian?

1 2 3 4

not at all important rather not important quite important very important

# He/she should be familiar with Hungarian culture.

How important is it for someone to be called Hungarian?

1 2 3 4

not at all important rather not important quite important very important

# He/she should speak Hungarian.

How important is it for someone to be called Hungarian?

1 2 3 4

not at all important rather not important quite important very important

# Thank you! Next, questions about children, birth and childbirth.

1. **Have** **you** **had** **a** **baby?**

If you have had a child who has unfortunately passed away, please consider them as well.

1. – no → SKIP
2. – yes

# Number of children born alive:

Please, enter a number

# What year was your (youngest) child born?

Please indicate with a year!

# Where was your (youngest) child born?

1. – In Hungary, in a state, municipal or university hospital or clinic
2. – In Hungary, in a private hospital or clinic
3. – At home
4. – Abroad
5. – Other place

# Was the birth of your (youngest) child a paternal birth?

1. – Yes
2. – No, because the mother didn’t want it / wouldn’t have wanted
3. – No, because the father didn’t want it / wouldn’t have wanted
4. – No, because hospital regulations didn’t allow
5. – No, because he was busy or couldn't get there
6. – For other reasons, but not

# When your (youngest) child was born, how satisfied were you with your obstetric care?

1. – I was completely satisfied →JUMP
2. – I was rather satisfied →JUMP
3. – Rather not satisfied
4. – I was not satisfied at all

# What is the main reason for your dissatisfaction?

1. – Inadequate human treatment -> INVITATION FOR AN INTERVIEW
2. – Inadequate medical, professional treatment
3. – Unexpected medical complication occured
4. – Other reason

The research will involve interviews with people who were dissatisfied with the human treatment they received in the maternity ward. If you would like to tell your story, please email us at [szalma.ivett@tk.hu](mailto:szalma.ivett@tk.hu)!

# Do you think that better services are provided in maternity hospitals for, those…:

- 1. **…who** **has** **better** **financial** **situation.**

Do you think you get better service in obstetrics?

1 2 3 4

no, worse no, the same yes, better yes, much better

# …who has adequate knowledge of hospital procedures.

Do you think you get better service in obstetrics?

1 2 3 4

no, worse no, the same yes, better yes, much better

# …who communicates well with the staff of the institution.

Do you think you get better service in obstetrics?

| 1 | 2 | 3 | 4 |
| --- | --- | --- | --- |
| no, worse | no, the same | yes, better | yes, much better |

# …who pays hospital staff a gratuity.

Do you think you get better service in obstetrics?

1 2 3 4

no, worse no, the same yes, better yes, much better

# …who has a relative is present during the birth.

Do you think you get better service in obstetrics?

1 2 3 4

no, worse no, the same yes, better yes, much better

# …who member of an ethnic or national minority.

Do you think you get better service in obstetrics?

1 2 3 4

no, worse no, the same yes, better yes, much better

# … who has a private doctor.

Do you think you get better service in obstetrics?

1 2 3 4

no, worse no, the same yes, better yes, much better

# How much do you agree that a woman about to give birth without a health problem should opt for a planned caesarean section?

1. – I totally disagree
2. – I rather disagree
3. – I agree and disagree
4. – I rather agree
5. – I totally agree

# Thank you very much! The following questions are about abortion.

1. **Do** **you** **think** **public** **education** **has** **given** **you** **enough** **knowledge** **about** **childbearing,** **human** **reproduction** **and** **sexual** **health?**
2. – I have not received such knowledge at all
3. – Rather no
4. – Rather yes
5. – I have received fully sufficient knowledge

# Do you think you have sufficient knowledge about childbearing, human reproduction and sexual health?

1. – Yes, I have sufficient knowledge
2. – No, I just have a limited knowledge
3. – No, I don’t have any knowledge about this
4. **What** **do** **you** **think** **is** **the** **ideal** **age** **for** **a** **woman** **to** **become** **a** **mother?**

Please give your answer with an age

1. **What** **do** **you** **think** **is** **the** **ideal** **age** **for** **a** **man** **to** **become** **a** **father?**

Please give your answer with an age

# After how old do you think a woman is too old to consider having children??

Please give your answer with an age

1. **After** **how** **old** **do** **you** **think** **a** **man** **is** **too** **old** **to** **consider** **becoming** **a** **father?**

Please give your answer with an age

# Before how old do you think a woman is too young to consider having children?

Please give your answer with an age

# Before how old do you think a man is too young to consider becoming a father?

Please give your answer with an age

# In which of the following cases should abortion be allowed in the first 3 months of pregnancy?

Tick all the cases where you think abortion should be allowed. If none, do not tick any of them

1. – If the woman's life or health is in danger.
2. – If the child is developing abnormally.
3. – If the woman would find it difficult to care for the child for personal reasons.
4. – In all cases where the woman asks for it, because no one should be forced to continue a pregnancy against her will.

# Do you think abortion regulation in Hungary should be changed?

1. – Yes, the current rules should be tightened.
2. – Yes, the current rules should be relaxed.
3. – No, it is properly regulated.
4. – I don't know, I don't know the regulation of abortion.

# In the following situations, how acceptable do you think it is for the pregnant woman in the story to choose abortion?

- 1. **During** **pregnancy,** **it** **is** **proven** **that** **the** **foetus** **will** **be** **born** **with** **a** **severe** **disability.**

In this case, do you think abortion is acceptable?

| 1 | 2 | 3 | 4 |
| --- | --- | --- | --- |
| not acceptable at all | rather not acceptable | rather acceptable | totally acceptable |

# During pregnancy, medical examinations confirm that pregnancy and childbirth a serious risk to the health of the pregnant woman.

In this case, do you think abortion is acceptable?

| 1 | 2 | 3 | 4 |
| --- | --- | --- | --- |
| not acceptable at all | rather not acceptable | rather acceptable | totally acceptable |

# A female member of a young Roma couple become pregnant, but their financial situation does not allow them to raise a child.

In this case, do you think abortion is acceptable?

| 1 | 2 | 3 | 4 |
| --- | --- | --- | --- |
| not acceptable at all | rather not acceptable | rather acceptable | totally acceptable |

# Fetus conceived through rape.

In this case, do you think abortion is acceptable?

| 1 | 2 | 3 | 4 |
| --- | --- | --- | --- |
| not acceptable at all | rather not acceptable | rather acceptable | totally acceptable |

# A 42-year-old mother of 3 gets pregnant by her husband, but no longer wants more children.

In this case, do you think abortion is acceptable?

| 1 | 2 | 3 | 4 |
| --- | --- | --- | --- |
| not acceptable at all | rather not acceptable | rather acceptable | totally acceptable |

# A 17-year-old girl still at school gets pregnant by her partner of the same age.

In this case, do you think abortion is acceptable?

| 1 | 2 | 3 | 4 |
| --- | --- | --- | --- |
| not acceptable at all | rather not acceptable | rather acceptable | totally acceptable |

# A 25-year-old girl becomes pregnant by her casual partner, but he leaves her life and she can't rely on him at all to care for and raise the child.

In this case, do you think abortion is acceptable?

| 1 | 2 | 3 | 4 |
| --- | --- | --- | --- |
| not acceptable at all | rather not acceptable | rather acceptable | totally acceptable |

# A female member of a young couple becomes pregnant, but their financial situation does not allow them to raise a child at all.

In this case, do you think abortion is acceptable?

| 1 | 2 | 3 | 4 |
| --- | --- | --- | --- |
| not acceptable at all | rather not acceptable | rather acceptable | totally acceptable |

# In which cases do you think the state funds abortion?

You can specify more option!

1. – Under no circumstances.
2. – In any case.
3. – Based on financial need.
4. – If it is based on a medical recommendation.
5. – If the couple has used a proven effective method of contraception and the pregnancy occurred despite this.
6. – If the woman is not in a relationship.
7. – If the pregnancy is the result of violence.
8. – If the woman already has at least three children.
9. – If the woman is too old to have children.
10. – If someone is too young to have children.

# What do you think about doctors being free to choose not to perform abortions (e.g. for conscientious or religious reasons)?

1. – I totally disagree, as this could violate women's right to abortion.
2. – I will only accept it if the doctor finds another professional to perform the abortion instead of himself.
3. – I accept in any case: the patient must find a specialist, even in private care, who can do it.

# Do you have personal experience of abortion (whether through a partner, close friend, friend, relative, or you have experienced it)?

1. – no
2. – yes

In the course of the research we would like to interview people who have had experience with abortion. If you would like to tell your story or the story of your (former) partner, please email us at [szalma.ivett@tk.hu](mailto:szalma.ivett@tk.hu)!

1. **Do** **you** **think** **the** **use** **of** **abortion** **pills** **should** **or** **should** **not** **be** **allowed** **in** **Hungary?** **(**Abortion pill: a preparation that allows abortion without medical intervention, which has been shown to be much less stressful for women than an induced abortion)
2. – Yes, it should be allowed.
3. – No, it shouldn’t be allowed.

# Thank you very much! The following questions are about contraception.

1. **What** **do** **you** **think** **public** **education** **has** **given** **enough** **knowledge** **about** **contraception?**
2. – I didn’t receive any such knowledge at all.
3. – Rather no.
4. – Rather yes.
5. – I received fully sufficient knowledge.

# What do you think do you have enough knowledge about contraception?

1. – Yes, I have enough knowledge
2. – No, I just have a limited knowledge
3. – No, I just have little knowledge

# Where does your knowledge of contraception come from, primarily?

You can specify multiple sources!

1. – From school
2. – From a doctor
3. – From parents
4. – From friends
5. – From the media (TV, newspaper)
6. – From Internet
7. – Other methods, namely:…

# Which of the following methods of contraception do you think is the most effective?

1. – Natural methods (calendar, interrupted intercourse)
2. – Intrauterine hormonal contraceptive device
3. – Intrauterine hormone-free contraceptive device
4. – Contraceptive pill, vaginal ring
5. – Condom, diaphragm
6. – Other methods, namely:…

# Which do you think is a greater health risk for a woman?

1. – Delivery of pregnancy, childbirth
2. - Use of hormonal contraception for 5 years
3. – Intrauterine hormone-free contraceptive device
4. – The above are associated with a similar level of risk

# Do you think a woman who has been using an intrauterine contraceptive device for 5 years will find it more difficult to get pregnant in the future after removing the device?

1. – No
2. – Yes

# Which do you think is a greater health risk for a woman?

1. – Abortion in the first 3 months of pregnancy
2. - Delivery of pregnancy, childbirth
3. – Both involve a similar level of risk

# Do you think that a woman who has an abortion in the first 3 months of an unplanned pregnancy is at greater risk of serious psychological/ mental health problems than if she continues the pregnancy?

1. – No
2. – Yes

# Do you think that a woman who has an abortion in the first 3 months of pregnancy is more likely to develop breast cancer than if she continues the pregnancy?

1. – No
2. – Yes

# o you think that a woman who has an abortion in the first 3 months of an unplanned pregnancy will find it more difficult to get pregnant in the future?

1. – No
2. – Yes

# How do you think access to the post-event pill (emergency contraceptive pill) should be made possible?

1. – As easily as possible, either in pharmacies or drugstores, without a prescription, available to anyone.
2. – As easily as possible, either in pharmacies or drugstores, without a prescription, but only for adults or under 18 accompanied/supervised by a parent.
3. – Prescription only, after medical consultation.
4. – I would not allow it at all.

# Who do you think should have access to female artificial sterilisation?

Female artificial sterilisation is a surgical procedure in which the fallopian tubes are closed, cut or tied to prevent the egg and the sperm from meeting. Which of the following answers is closest to your opinion?

1. – No one.
2. – For all female over 18, who wish to claim.
3. – For all women over 18 who have already given birth to at least one child and wish to claim.
4. – For any woman who has already given birth to at least 3 children and wish to calim.
5. – For all women over a certain age (for example, those over 40).
6. – None of the answer options are close to my opinion.

# Who do you think should have access to male artificial sterilisation?

Male artificial sterilisation is a minor surgical procedure in which the testicular ducts are cut or blocked to prevent sperm from being expelled. Which of the following answers is closest to your opinion?

1. – No one.
2. – For all men over 18, who wish to claim.
3. – For all men over 18 who already have at least one child and wish to claim.
4. – For all men who already have 3 children and wish to claim.
5. – For all men over a certain age (for example, those over 40).
6. – None of the answer options are close to my opinion.

# Do you (still) want to have children?

1. – Yes, within 1 year→ SKIP
2. – Yes, within 5 years
3. - Yes, but in more than 5 years
4. – I would not like to.
5. – For health reasons or because of my age, I can no longer have children → SKIP

# Are you or your partner using any form of protection against an unwanted pregnancy?

1. – No → SKIP
2. – Yes

# How to protect against an unwanted pregnancy?

1. – Natural methods (calendar method, interrupted intercourse).
2. - Intrauterine hormonal contraception.
3. - Intrauterine contraception without hormones.
4. - Contraceptive pill, vaginal ring.
5. - Condom, diaphragm.
6. – Artificial sterilization has occurred.
7. – By other methods.

# Have you ever been unprotected during a casual sexual relationship?

1. – No, because I have not had that relationship.
2. – No, because I always use protection.
3. – Yes, it rarely happened.
4. - Yes, it happened often.
5. - Yes, I never used protection.

# Do you think that the use of contraceptives (e.g. condoms, diaphragms, contraceptive pills, intrauterine contraceptives) should be financed by the state?

Which of the following answers is closest to your opinion?

1. – Yes, absolutely for everyone.
2. – Yes, but only on the basis of need.
3. - Yes, but only for minors.
4. - Yes, but only for those under 25.
5. – No.

# Do you think you have received enough information about infertility treatment in public education?

1. – I have not received any such knowledge at all
2. - Rather not
3. - Rather yes
4. - I received fully sufficient knowledge

# Do you think you have enough knowledge about infertility procedures?

1. - Yes, I have sufficient knowledge
2. - No, I have little knowledge
3. - No, I do not have any knowledge

# What is your primary source of knowledge about infertility procedures?

You can specify multiple sources!

1. – From school
2. - From a doctor
3. - From parents
4. - From friends
5. - From the media (TV, newspaper)
6. - From the Internet
7. - From other sources, namely...

# Do you consider the following infertility procedures acceptable?

- 1. **Methods** **to** **promote** **ovulation** **(hormonal** **preparations).**

Is it acceptable to you?

| 1 | 2 | 3 | 4 | 9 |
| --- | --- | --- | --- | --- |
| not acceptable at all | rather not acceptable | rather acceptable | perfectly acceptable | I do not know |

# Insemination (artificial sperm insertion).

Is it acceptable to you?

| 1 | 2 | 3 | 4 | 9 |
| --- | --- | --- | --- | --- |
| not acceptable at all | rather not acceptable | rather acceptable | perfectly acceptable | I do not know |

# IVF (in vitro fertilization).

Is it acceptable to you?

| 1 | 2 | 3 | 4 | 9 |
| --- | --- | --- | --- | --- |
| not acceptable at all | rather not acceptable | rather acceptable | perfectly acceptable | I do not know |

# Use of donor sperm in cases of male infertility.

Is it acceptable to you?

| 1 | 2 | 3 | 4 | 9 |
| --- | --- | --- | --- | --- |
| not acceptable at all | rather not acceptable | rather acceptable | perfectly acceptable | I do not know |

# Use of donor eggs in cases of female infertility.

Is it acceptable to you?

| 1 | 2 | 3 | 4 | 9 |
| --- | --- | --- | --- | --- |
| not acceptable at all | rather not acceptable | rather acceptable | perfectly acceptable | I do not know |

# Which would you choose / which would you have chosen in the event of infertility?

1. – Seeking medical assistance
2. - Adoption
3. - Childlessness
4. - Other solution, namely:….

# What medical help would you seek?

Indicate all treatments you would undertake!

1. – Surgery
2. – Insemination (artificial insertion of sperm)
3. – Lombic treatment (IVF)

# Who do you think should be the beneficiaries of state funding for IVF procedures?

You can specify multiple response options!

1. – It should be available to anyone, without limitation in number.
2. - For the socially deprived.
3. - For married people.
4. - For younger couples (e.g. under 40).
5. - It should not be funded for anyone.

# Do you think that the state should also finance medicines for the use of IVF?

1. – Yes, absolutely
2. – Only part of it
3. – Only on the basis of need
4. – Only for young people
5. – No

# Do you think it should be possible for lesbian women to have access to IVF?

1. – No
2. – Yes

# Do you think it should be possible for single women to have access to IVF procedures?

1. – No
2. – Yes

# Do you think it should be possible for older women (over 40) to have access to IVF?

1. – No
2. – Yes

# Are you for or against surrogacy, i.e. a woman carrying someone else's child?

1. – I am totally against -> SKIP
2. – I consider it acceptable in certain cases
3. - I consider it acceptable in its entirety

# Please select in which of the following cases you consider surrogacy to be acceptable!

1. – If there are medical reasons.
2. - If two gay men want to have children.
3. - If someone is afraid of giving birth.

# In the case of surrogacy, who would you consider the mother of the unborn child?

1. – Who gives birth to the child.
2. – Whom the oocyte comes.

# Thank you very much! In order for the results to be aggregated and interpreted by researchers, we just need some background information from you.

1. **What** **is** **your** **gender?**
2. – Male
3. – Female
4. – Other

# What year were you bor?

Please indicate your answer with a year!

# In which municipality do you live?

1. – Capital city
2. - County seat
3. - Other city
4. - Village, parish

# What is your official marital status?

1. – Unmarried, single
2. – Married, living with spouse 🡪SKIP
3. – Living with registered companion 🡪SKIP
4. – Married but not living with spouse
5. – Widowed
6. – Divorced

# Which statement is true about your situation?

1. – You live with your companion
2. – You have a partner but do not live with him/her
3. – You don’t have a partner

# What is your highest level of education?

1. – 8 or fewer years of primary school
2. – Vocational Training, vocational school
3. – Technical college, technical institute
4. – High school
5. – College / university bachelor's degree
6. – University / university master's degree
7. – PhD degree

# What was your mother's highest level of education when you were 14 years old?

1. – 8 or fewer years of primary school
2. – Vocational Training, vocational school
3. – Technical collage
4. – High school
5. – College / university bachelor's degree
6. – University / university master's degree
7. – PhD degree

# What is your main occupation?

If there are several, choose the most typical one!

1. – Employee – full-time
2. – Employee – part-time
3. – Contractor
4. – Unemployed
5. – Homemaker
6. – In receipt of maternity benefits (csed, gyed, gyes, gyet)
7. – Studying full-time
8. – Old-age pensioner
9. – Disability pensioner, receiving invalidity benefit
10. – Other

# Which description best approximates your household's current income situation?

1. – We live comfortably on our current income.
2. – We get by on our current income.
3. – We have difficulty living on our current income.
4. – We have a very hard time living on our current income.

# Do you belong to a religious denomination? If so, which one?

1. – Not belonging to a denomination
2. – Roman Catholic
3. – Greek Catholic
4. – Reformed
5. – Evangelical
6. – Orthodox
7. – Israelita
8. – Buddhist
9. – Belongs to other denominations

# Which of the following statements would best describe yourself?

1. – I’m religious, I follow the teachings of the Church
2. – I’m religious in my own way
3. – I cannot say whether I am religious
4. – I’m not religious
5. **Do** **you** **have** **a** **pet?**
6. – no → SKIP
7. – yes

# What kind of pets do you have?

(Select from the list all the ones you have)

- - 1. – dog
    2. – cat
    3. – fowl (e.g. parrot, finch)
    4. – ornamental fish
    5. – rabbit

1. – rodent (e.g. hamster, guinea pig, mouse)
2. – reptile (e.g. lizard, snake)
3. – other

# If you have any comments or opinions on the topics covered in the questionnaire, please let us know below.

Thank you very much for your responses, which contributed to the success of the survey of the “Momentum” Reproductive Sociology Research Group! For further information on the follow-up and results of the survey, please contact Ivett Szalma, PhD, Research Professor, at [szalma.ivett@tk.hu.](mailto:szalma.ivett@tk.hu)

Your reply will be rewarded with a 2000 HUF EMAG voucher, which will be sent to your e-mail address. Please submit the completed questionnaire!
